# Active Expression of Human Hyaluronidase PH20 and Characterization of Its Hydrolysis Pattern

**DOI:** 10.3389/fbioe.2022.885888

**Published:** 2022-05-13

**Authors:** Bo Pang, Jing He, Weijiao Zhang, Hao Huang, Yang Wang, Miao Wang, Guocheng Du, Zhen Kang

**Affiliations:** ^1^ School of Food Science and Technology, Jiangnan University, Wuxi, China; ^2^ The Key Laboratory of Carbohydrate Chemistry and Biotechnology, Ministry of Education, School of Biotechnology, Jiangnan University, Wuxi, China; ^3^ The Science Center for Future Foods, Jiangnan University, Wuxi, China

**Keywords:** *Pichia pastoris*, human hyaluronidase, PH20, domain truncation, heterologous expression, hydrolysis pattern

## Abstract

Hyaluronidases are a group of glycosidases catalyzing the degradation of hyaluronic acid (HA). Because of the advantages of effectively hydrolyzing the HA-rich matrix and low immunogenicity, human hyaluronidase PH20 (hPH20) is widely used in the medical field. Here, we realized the active expression of recombinant hPH20 by *Pichia pastoris* under a methanol-induced promoter P_AOX1_. By optimizing the composition of the C-terminal domain and fusing protein tags, we constructed a fusion mutant AP_2_-△491C with the extracellular hyaluronidase activity of 258.1 U·L^−1^ in a 3-L bioreactor, the highest expression level of recombinant hPH20 produced by microbes. Furthermore, we found recombinant hPH20 hydrolyzed the β-1,4 glycosidic bonds sequentially from the reducing end of o-HAs, with HA_6_^NA^ as the smallest substrate. The result will provide important theoretical guidance for the directed evolution of the enzyme to prepare multifunctional o-HAs with specific molecular weights.

## Introduction

Hyaluronidases (Hyals) are a large class of glycosidases that predominately catalyze the degradation of hyaluronic acid (HA) (Fronza et al., 2016; Khan et al., 2018). Due to their functions of anesthetic assistance, reducing intraocular pressure, facilitating drug absorption, and resistance to tumor signaling, Hyals have been widely used in medical fields (Locke et al., 2019; Feng et al., 2021; and Knowles et al., 2021). Based on the differences in their catalytic mechanisms and end products, Hyals are classified into three families (Bookbinder et al., 2006; El-Safory et al., 2010; and Kang et al., 2018): hyaluronate 4-glycanohydrolases (EC 3.2.1.35, mammalian hyaluronidases, hydrolyzing the β-1,4 glycosidic bonds of HA, and furnishing tetrasaccharide as the main product), hyaluronate 3-glycanohydrolases (EC 3.2.1.36, leech hyaluronidases, hydrolyzing the β-1,3 glycosidic bonds of HA, and generating tetra- and hexasaccharide end products), hyaluronate lyases (EC 4.2.2.1, bacterial lyases, degrading HA by a β-elimination reaction, and yielding unsaturated disaccharides as the main products). To date, Hyals from animal tissues, leeches, venoms, and various microorganisms have been studied (Tan and Ponnudurai, 1992; Oettl et al., 2003; Rigden and Jedrzejas, 2003; Yang and Lee, 2006; Jin et al., 2014).

Human hyaluronidase PH20 (hPH20), a glycosylphosphatidylinositol (GPI)-anchored membrane protein in mammalian sperm, is a widely recognized hyaluronidase (Cherr et al., 2001). hPH20 could effectively hydrolyze the HA-rich matrix of the oocyte to facilitate the penetration of the sperm. Thus, hPH20 has great potential applications in medical fields (Hong et al., 2019; Usmani et al., 2019; Connor et al., 2020). In past years, the successful expression of recombinant hPH20 in insect or Chinese hamster ovary (CHO) cells has been reported (Frost, 2007; Hofinger et al., 2007); however, the disadvantages such as complex operation, long culture cycle, and high cost restrict its applications. By contrast, *Pichia pastoris* gives higher expression levels of a wide variety of recombinant enzymes and is generally regarded as easier, faster, and less expensive than insect and mammalian expression systems (Cereghino and Cregg, 2000). In a previous study, Chen et al. have realized the constitutive expression of recombinant hPH20 in *P. pastoris*, and even the activity was merely 2 U·L^−1^ (Chen et al., 2016). To meet the industrial requirement, the expression of recombinant hPH20 needs a considerable improvement. Furthermore, to date, the hydrolysis pattern of recombinant hPH20 on HA still remains unclear.

In this study, we analyzed the hPH20 sequence and achieved the comparatively high expression of recombinant hPH20 in *P. pastoris* with the methanol-induced promoter P_AOX1_. Through the optimization of the C-terminal domain and application of protein fusion tags, we successfully constructed a fusion mutant AP_2_-△491C with the extracellular hyaluronidase activity of 258.1 U·L^−1^ in a 3-L bioreactor, which was the highest value from microbes to date. Moreover, hydrolysis pattern analysis results demonstrate that recombinant hPH20 hydrolyzes the β-1,4 glycosidic bonds sequentially from the reducing end of o-HAs with HA_6_^NA^ as the smallest substrate.

## Materials and Methods

### Strains, Plasmids, and Reagents

*E. coli* JM109 and plasmid pPIC9K were used for gene cloning and propagation procedures. *P. pastoris* GS115 was used as a host for the expression of recombinant hPH20 and its mutants. PrimeSTAR Max DNA Polymerase, T4 DNA ligase, T4 polynucleotide kinase, restriction endonucleases, and protein markers were purchased from TaKaRa (Dalian, China). Ampicillin, G418 sulfate, and plasmid Mini Prep Kit were purchased from Sangon Biotech (Shanghai, China). The gel extraction kit and 10% Bis-Tris protein gel were purchased from Thermo Scientific (Shanghai, China). Hyaluronidase from bovine testicular tissue (BTH) was obtained from Sigma-Aldrich (St Louis, MO, United States). Other chemicals were obtained commercially and were of reagent grade.

### Medium

Luria–Bertani (LB) medium (10 g L^−1^ tryptone, 10 g L^−1^ NaCl, 5 g L^−1^ yeast extract, and pH 7.0) was used for the cloning experiment. The MD plate [20 g L^−1^ glucose, 13.4 g L^−1^ yeast nitrogen base (YNB), and 20 g L^−1^ agar] was used for screening *P. pastoris* GS115 recombinants. YPD medium (10 g L^−1^ yeast extract, 20 g L^−1^ tryptone, and 20 g L^−1^ glucose), BMGY medium (10 g L^−1^ yeast extract, 20 g L^−1^ tryptone, 100 mM potassium phosphate, 13.4 g L^−1^ YNB, 4 × 10^–4^ g L^−1^_D_-biotin, and 10 g L^−1^ glycerol), and BMMY medium [10 g L^−1^ yeast extract, 20 g L^−1^ tryptone, 100 mM potassium phosphate, 13.4 g L^−1^ YNB, 4 × 10^–4^ g L^−1^_D_-biotin, and 1% (*v/v*) methanol] were used for flask cultures. Trace metal solution (PTM1, 3 g L^−1^ MnSO_4_·H_2_O, 6 g L^−1^ CuSO_4_·5H_2_O, 0.2 g L^−1^ MoNa_2_O_4_·2H_2_O, 65 g L^−1^ FeSO_4_·7H_2_O, 0.5 g L^−1^ CoCl_2_, 20 g L^−1^ ZnCl_2_, 0.09 g L^−1^ KI, 0.02 g L^−1^ H_3_BO_3_, 0.2 g L^−1^_D_-biotin, and 5 ml L^−1^ H_2_SO_4_) and BSM (18 g L^−1^ K_2_SO_4_, 14.9 g L^−1^ MgSO_4_·7H_2_O, 4.13 g L^−1^ KOH, 40 g L^−1^ glycerol, 27 ml L^−1^ H_3_PO_4_, and 0.93 g L^−1^ CaSO_4_ with 4.4 ml L^−1^ PTM1) were used for fed-batch fermentation.

### Genetic Operations

According to the codon bias of *P. pastoris*, we optimized the codons of the *hPH20* gene carrying the His × 6-tag coding sequence. The codon-optimized DNA fragment was cloned to restriction sites *EcoR* I and *Not* I in pPIC9K to obtain plasmid pPIC9K-*hPH20*. The primers (Supplementary Table S1) were used for cloning the C-terminal truncated mutants. The PCR products were extracted using the gel extraction kit. The extracted product, T4 DNA ligase, T4 polynucleotide kinase, and T4 DNA ligase buffer were added to a PCR tube at a ratio of 7:1:1:1 (v/v), incubated at 16°C for 12 h, and then transformed into *E*. *coli* JM109 for sequencing. Plasmids with the correct sequences were linearized by *Sal* I and transformed into competent *P. pastoris* GS115 cells *via* electroporation (voltage: 2000 V, capacitance: 25 *μ*F, resistance: 200 Ω, and cuvette: 2 mm) using a GenePulser Xcell™ apparatus purchased from Bio-Rad (Hercules, United States). The transformants were screened on MD plates, and the selection of multi-copy cells was performed on YPD plates with 4 g L^−1^ G418 sulfate. The gene copy numbers of all recombinant strains were determined by real-time PCR, as described previously (Kang et al., 2016). Clones harboring nine copies of the target gene of each construct were selected.

We had three protein fusion tags (AP_2_, HL_28_, and Sumo-tag) synthesized before the N terminal of the truncated mutant △491C to obtain three recombinant expression plasmids: pPIC9K-*ap*_*2*_-*△491C*, pPIC9K-*hl*_*28*_-*△491C*, and pPIC9K-*sumo*-*△491C*. Then, we transformed and screened the *P. pastoris* GS115 recombinants as before.

### Shake Flask Culture and Fed-Batch Fermentation

*P. pastoris* GS115 strains carrying recombinant hPH20 were inoculated in 250-ml flasks supplied with 50 ml YPD medium and grown at 30°C and 220 rpm for 24 h. Then, the yeast strains were transferred to a BMGY medium with a 10% (*v/v*) inoculum and cultivated at 30°C and 220 rpm until the OD_600_ value reached 6. The yeast strains were collected and washed with 0.9% NaCl three times, then transferred to 50 ml BMMY medium to induce the expression at 30°C and 220 rpm. All the flasks were added with 1% (*v/v*) methanol every 24 h, and experiments were carried out in triplicate.

Fed-batch fermentation was performed in a 3-L bioreactor (BXBIO, Shanghai, China) using a 900 ml BSM. A 10% (*v/v*) inoculum was obtained after cultivating the strains at 30°C and 220 rpm for 24 h. The initial fermentation parameters were set at 30°C, pH 5.0, 2.0 vvm, and 220 rpm. After glycerol exhaustion indicated by a sudden increase in dissolved oxygen, 50% (*w/v*) glycerol supplemented with 1.2% (*v/v*) PTM1 was fed at a constant rate of 25 ml L^−1^ h^−1^ for 10 h. Methanol supplemented with 1.2% (*v/v*) PTM1 was fed at a constant rate of 7 ml L^−1^ h^−1^ to induce the expression of recombinant hPH20. The culture broth was collected every 12 h for analysis of cell growth and hyaluronidase activity.

### Western Blot Analysis of Recombinant hPH20 Expression

For the detection of recombinant hPH20 expression, samples were harvested by centrifugation (9,000 × *g* for 10 min at 4°C). The supernatant was mixed with 4 × Protein Native PAGE Loading Buffer (TaKaRa, Dalian, China) and heated at 100°C for 10 min. SDS-PAGE was performed by 10% Bis-Tris Protein Gels in MES running buffer at 100 V. Then, SDS-PAGE gel was electroblotted to PVDF transfer membrane (PerkinElmer, Shanghai, China) using a transfer buffer containing 20% methanol, 15.1 g L^−1^ glycine, and 3.0 g L^−1^ Tris. Blotted membrane was blocked with QuickBlock™ Blocking Buffer (Beyotime Biotechnology, Shanghai, China) for 1 h. For immunodetection, His-tag Mouse Monoclonal Antibody (Beyotime Biotechnology, Shanghai, China) diluted 1: 1,000 with TBS (2.4 g L^−1^, pH 7.6 Tris buffer containing 8.0 g L^−1^ NaCl) was used as a primary antibody. After washing with TBST (2.4 g L^−1^, pH 7.6 Tris buffer containing 8.0 g L^−1^ NaCl and 0.1% Tween-20) for 30 min, a 1:1,000 dilution of the secondary antibody, HRP-labeled Goat Anti-Mouse lgG (H + L) (Beyotime Biotechnology, Shanghai, China) was added and incubated at 4°C with gentle shaking for 1 h. After washing with TBST for 30 min, DAB Horseradish Peroxidase Color Development Kit (Beyotime Biotechnology, Shanghai, China) was used to develop the color on the membrane.

### Determination of Recombinant hPH20 Activity

Recombinant hPH20 activity was determined using the 3,5-dinitrosalicylic acid (DNS) method to measure the reducing sugar released from hyaluronic acid (Asteriou et al., 2001). In this study, 100 μl of 1.25 mg ml^−1^ hyaluronic acid in 100 mM, pH 5.0 acetate buffer was mixed with 100 μl of enzyme sample and incubated at 37°C for 30 min. Thereafter, 200 μl of DNS was added to the standard reaction system to terminate the reaction. The reaction was boiled in a water bath at 100°C for 6 min and then cooled on ice immediately. The reaction system with inactivated enzyme was used as a control. Enzyme activity (U) was defined as the amount of enzyme that released 1 μmol of reducing sugar [equivalent to *N*-acetyl-glucosamine (NAG)] per minute under specified assay conditions. All values were expressed as the mean ± standard deviation (SD ≤ 5%) of three independent experiments.

### Hydrolysis of Recombinant hPH20 on Different Glycosaminoglycan Substrates

An aliquot of 100 μl of 1.25 mg ml^−1^ different glycosaminoglycan substrates [HA, chondroitin sulfate type A (CSA), chondroitin sulfate type C (CSC), and heparin (HP)] in 100 mM, pH 5.0 acetate buffer was mixed with 100 μl of enzyme sample and incubated at 37°C for 30 min. Thereafter, 200 μl of DNS was added to the standard reaction system to terminate the reaction. Recombinant hPH20 activity was quantified as described earlier.

### Preparation of HA_2n_^NA^ o-HAs

The enzymatic properties and hydrolysis process of LHyal have been studied (Jin et al., 2014). To prepare HA_2n_^NA^ o-HAs (HA_4_^NA^, HA_6_^NA,^ and HA_8_^NA^), 1 g of HA was fully dissolved in 100 ml of distilled water and pre-incubated at 37°C. Afterward, 3000 U·ml^−1^ of LHyal was mixed with the aforementioned HA solution and incubated at 37°C for 20 h, then LHyal was inactivated by boiling for 5 min. After high-speed centrifugation (9,000 × g for 30 min at 4°C) to remove inactivated enzyme, a HiPrep Q HP 16/10 column (column volume: 20 ml, GE Healthcare) was used to separate HA_2n_^NA^ o-HAs from the supernatant. The column was balanced by buffer A (0.02 M, pH 8.0 PBS) before eluting by a linear gradient of 0–100 mM NaCl (pH 8.0) for 3 CV under 3 ml min^−1^ flow rate.

Each fraction corresponding to a chromatographic peak was desalted using a Superdex 30 Increase 10/300 G L column and lyophilized to get the final products. Each HA_2n_^NA^ o-HA fraction was qualitatively analyzed by high-performance liquid chromatography–mass spectrometry (HPLC-MS) to confirm purity, as described previously (He et al., 2020).

### Hydrolysis of HA_2n_^NA^ o-HAs and Analysis of Hydrolysates

Recombinant hPH20 was purified using a Ni-NTA column (GE Healthcare, Shanghai, China). An aliquot of 100 μl of recombinant hPH20 was mixed with 80 μl of purified o-HAs (HA_4_^NA^, HA_6_^NA^, and HA_8_^NA^), respectively, and incubated at 37°C for 8 h; then recombinant hPH20 was inactivated by boiling for 5 min. To remove the salt, 180 μl of methanol was added to the reaction system, mixed gently, and then centrifuged at 12,000 rpm for 10 min. Electrospray ionization-mass spectrometry (ESI-MS) spectra were acquired using a Quattro Premier XE system (Waters). Additionally, HPLC separation was performed using a Zorbax NH_2_ column (4.6 × 250 mm) at 40°C. The products were eluted with acetonitrile/water (75:25) at a flow rate of 1.0 ml min^−1^. The eluent was monitored by measuring the total ions in the mass range of *m/z* 100–600 in the negative mode.

## Results and Discussion

### Structure and Catalytic Domain Analysis of hPH20

The *hPH20* gene (GenBank accession number: NP_003,108.2) cloned from human sperm encodes a polypeptide of 511 amino acids. The molecular weight and pI value of hPH20 were estimated to be 58.4 kDa and 7.0, respectively. The conserved amino acids in hPH20 structural domains associated with substrate catalysis and glycosylation were deduced by the NCBI online protein blast tool: the active site Asp^146^ and Glu^148^, and the glycosylation site Asn^82^, Asn^166^, Asn^235^, Asn^254^, Asn^368^, and Asn^393^. Homology analysis revealed that hPH20 shows 86.1% identity with hyaluronidase from *Sapajus apella*, 75.1% with hyaluronidase from *Carlito syrichta*, and 70.0% with hyaluronidase from *Otolemur garnettii*, and 65.4% with hyaluronidase from *Ictidomys tridecemlineatus*.

**FIGURE 1:**
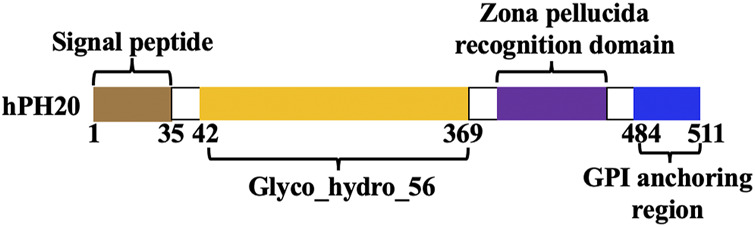
Structural domains of hPH20.

hPH20 was predicted to contain four structural domains by Protein Domain Analysis Tool (http://smart.embl-heidelberg.de). As shown in Figure 1, the domain compositions were signal peptide coding region (positions 1–35): found in the wild-type sequence of hPH20, related to transport and secretion; Glyco_hydro_56 (positions 42–369): N-terminal catalytic domain of hPH20, commonly found in GH56 protein; zona pellucida recognition domain (∼110 aa linked after Glyco_hydro_56): recognized by the zona pellucida of the oocyte, related to the binding of sperm and egg cells in fertilization; and GPI anchoring region (positions 484–511): a short hydrophobic structure in the C-terminal of hPH20, associated with the binding of protein to the cell membrane. Moreover, a protease cleavage site was predicted between Ser490 and Ala491 of the GPI anchoring region.

### Active Expression of Recombinant hPH20 With Its Truncated Variants

**FIGURE 2:**
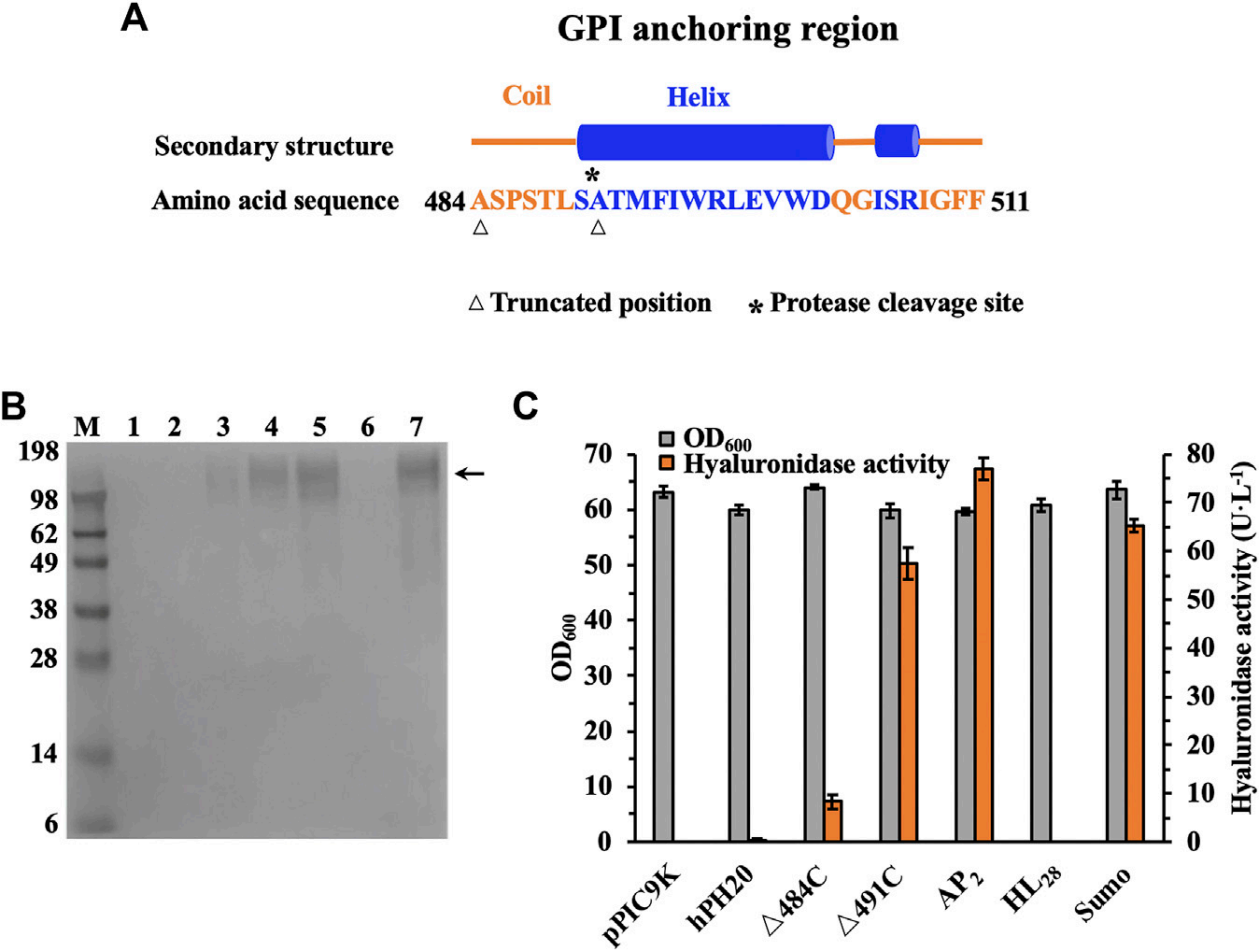
Expression and activity of recombinant hPH20, truncated mutants (^△^484C ^△^491C), and ^△^491C with different tags in the culture broth. **(A)** Secondary structure and sequence analysis of the GPI anchoring region. **(B)** Western blot analysis of the recombinant hPH20, truncated mutants (^△^484C ^△^491C), and ^△^491C with different tags. M: standard protein marker; 1: control; 2: recombinant hPH20; 3: ^△^484C; 4: ^△^491C; 5: AP_2_; 6: HL_28_; 7: Sumo. The truncated mutants (^△^484C ^△^491C) and ^△^491C with different tags are shown by the black arrow. **(C)** Hyaluronidase activity of recombinant hPH20, truncated mutants (^△^484C ^△^491C), and ^△^491C with different tags.

In consideration of deletion of functionally unnecessary domains could simplify the whole structure and improve the secretion efficiency and production of an enzyme (Kim et al., 2011; Duan and Wu, 2015), recombinant hPH20 was truncated and expressed with the *P. pastoris* expression system for characterization. Through the analysis of structural domains of recombinant hPH20, we found that the GPI anchoring region (positions 484–511) played a role in the binding of recombinant hPH20 and cell membrane, not related to the catalysis according to the description of domain function. Furthermore, a protease cleavage site existed between positions 490 and 491, indicating the amino acid sequence after position 490 may not affect the enzymatic properties of recombinant hPH20. Therefore, as shown in Figure 2A, we designed and constructed two C-terminal truncated mutants (hPH20△484C and hPH20△491C).

The molecular weights of secreted recombinant hPH20, hPH20△484C, and hPH20△491C were estimated to be 55.4, 52.2, and 52.8 kDa, respectively. However, we were not sure whether the protein bands of recombinant hPH20, hPH20△484C, and hPH20△491C in culture supernatants were present or not by SDS-PAGE analysis (Supplementary Figure S1). To confirm the expression of these variants, Western blot analysis with sensitive protein detection was carried out. As shown in Figure 2B, hPH20△484C and hPH20△491C in the culture supernatants were successfully detected with a very broad molecular mass of 62–198 kDa, which were higher than the calculated values. This may be due to the glycosylation that occurred in the endoplasmic reticulum. By contrast, the recombinant hPH20 in the supernatant was not detected. To identify the reason, we mixed the whole cell of *P. pastoris* GS115-pPIC9K-*hPH20* with HA and determined whether HA could be hydrolyzed. As shown in Supplementary Figure S2, the whole-cell hydrolyzed HA, indicating the recombinant hPH20 fixed on the cell membrane with the GPI anchoring region and its N-terminal catalytic domain, was exposed to the outside of the cell membrane, which accounted for not detecting recombinant hPH20 in the supernatant.

In parallel, the hyaluronidase activities of recombinant hPH20, hPH20△484C, and hPH20△491C in the culture broth were determined by using the DNS method. As shown in Figure 2C, the extracellular hyaluronidase activity of hPH20△491C reached 57.5 U·L^−1^, which was 6.8 times higher than that of hPH20△484C (8.4 U·L^−1^), indicating that the structure between 490 and 484 was indispensable for maintaining a high catalytic activity of recombinant hPH20. On the contrary, we did not observe any extracellular hyaluronidase activity of recombinant hPH20, indicating the C-terminal structure between 511 and 491 played a key role in the anchoring of recombinant hPH20 and cell membrane. The results of hyaluronidase activity determination were consistent with those of Western blot analysis.

To further improve the secretion efficiency and production, we optimized the composition of the C-terminal structure and constructed truncated mutants hPH20△507C, hPH20△502C, hPH20△497C, hPH20△489C, hPH20△487C, and hPH20△485C, comparing extracellular hyaluronidase activities of these mutants with that of hPH20△491C. As shown in Supplementary Figure S3, these mutants displayed lower hyaluronidase activity and expression levels than those of hPH20△491C. Therefore, we used hPH20△491C in the following experiments.

### Introduction of Protein Fusion Tags to Improve Recombinant hPH20 Expression

Protein fusion tags functioned to facilitate the expression of exogenous protein and increase the secretion efficiency of soluble protein (Moua et al., 2016; and Wang et al., 2019). For instance, Huang et al. achieved the extracellular high-level expression of LHyal in *P. pastoris* by fusing different N-terminal tags, the soluble expression of leech hyaluronidase (Huang et al., 2020). The secretion efficiency of *Candida antarctica* lipase B (Kim et al., 2015) and the (+)-zizaene synthase from *Chrysopogon zizanioides* (Hartwig et al., 2015) were also improved. Therefore, three commonly used protein fusion tags AP_2_, HL_28_, and Sumo-tag were fused to the N terminal of the variant hPH20△491C to assist the expression and secretion.

As shown in Figure 2B, fusion mutants AP_2_-△491C and Sumo-△491C in the culture broth were both detected by Western blot analysis, indicating the successful expression and secretion. By contrast, no HL_28_-△491C was documented, which might be due to the inhibitory effect of the protein tag HL_28_ on the expression of △491C. The hyaluronidase activities of AP_2_-△491C and Sumo-△491C were also comparatively analyzed. As shown in Figure 2C, the extracellular hyaluronidase activities of AP_2_-△491C and Sumo-△491C reached 76.9 U·L^−1^ and 65.4 U·L^−1^, respectively, 1.3 and 1.1 times higher than that of △491C, indicating the protein tags AP_2_ and Sumo could facilitate the expression and secretion of △491C. Significantly, the extracellular hyaluronidase activity of fusion mutant AP_2_-△491C in this study was 38.5 times higher than that of the previous report (Chen et al., 2016), suggesting the stronger promoter strength of P_AOX1_ than that of the constitutive promoter P_GAP_.

### Production of Recombinant hPH20 With High-Cell Density Fermentation

**FIGURE 3:**
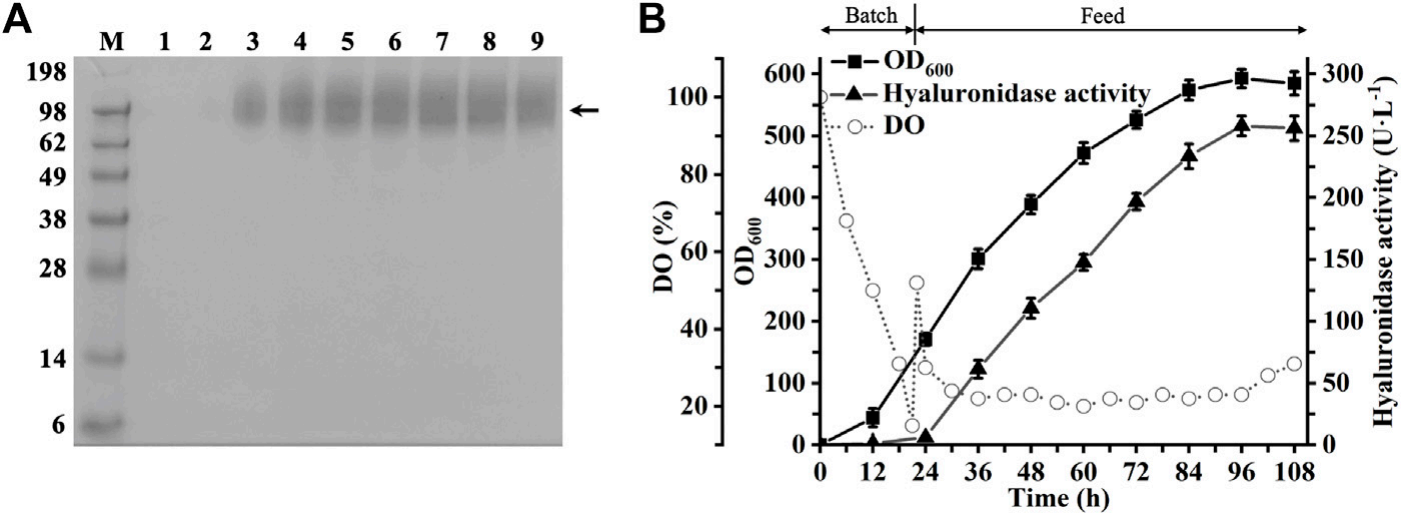
Fed-batch fermentation of recombinant strain *P. pastoris* GS115-pPIC9K-*ap*_*2*_-*△491C*. **(A)** Western blot analysis of the culture supernatants. M: standard protein marker; 1-9, samples were taken at 12, 24, 36, 48, 60, 72, 84, 96, and 108 h, respectively. The target proteins are shown by the black arrow. **(B)** Time course of cell growth, dissolved oxygen, and hyaluronidase activity in a 3-L bioreactor.

To evaluate the hyaluronidase-producing ability of recombinant *P. pastoris* GS115-pPIC9K-*ap*_*2*_-*△491C*, a high-cell fermentation was performed in a 3-L bioreactor. As shown in Figure 3B, the extracellular hyaluronidase activity of AP_2_-△491C reached 258.1 U·L^−1^ at 96 h, 3.4 times higher than that of flask cultures. Consistently, the Western blot analysis of the extracellular culture supernatant indicated the inducible expression and secretion of AP_2_-△491C during the whole process of fermentation (Figure 3A). To our knowledge, we achieved the highest expression level of recombinant hPH20 in studies of producing recombinant hPH20 by the microbial expression system. This result would lay a foundation for the large-scale preparation of recombinant hPH20 in future studies.

### Substrate Specificity of Recombinant hPH20

**FIGURE 4:**
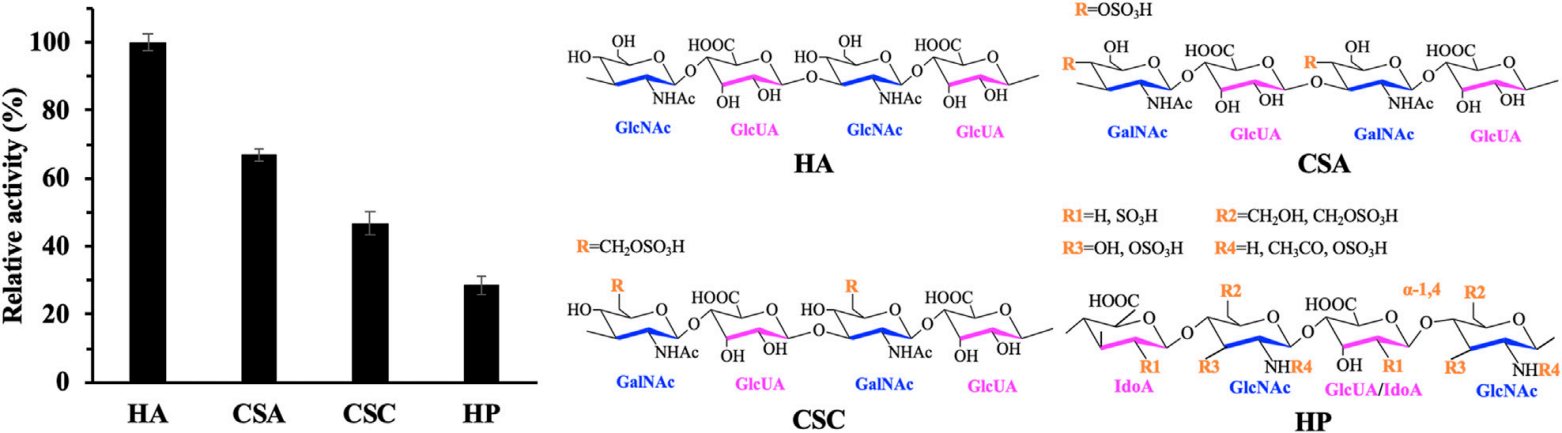
Hydrolysis activity of recombinant hPH20 on different glycosaminoglycan substrates.

To analyze the substrate specificity of recombinant hPH20, we chose different glycosaminoglycans (HA, CSA, CSC, and HP) as substrates to measure the hydrolytic activity of recombinant hPH20. As shown in Figure 4, HA, CSA, CSC, and HP could be hydrolyzed. Recombinant hPH20 showed the highest hydrolytic capacity toward substrate HA, 1.5, 2.1, and 3.5 times higher than those of substrates CSA, CSC, and HP. Compared with LHyal and BTH (Jin et al., 2014), recombinant hPH20 exhibited a broader substrate spectrum.

### Hydrolysis Pattern of Recombinant hPH20 on Substrate HA

In a previous study, it has been reported that recombinant hPH20 hydrolyzes the β-1,4 glycosidic bonds of the HA chain producing o-HAs with different molecular weights (Arming et al., 1997), but the hydrolysis pattern remains unknown. To solve this problem, we prepared HA_2n_^NA^ o-HAs (HA_4_^NA^, HA_6_^NA^, and HA_8_^NA^ shown in Supplementary Figure S4) by LHyal-catalyzed HA hydrolysis and analyzed the hydrolysates of o-HAs cleaved by recombinant hPH20.

**FIGURE 5:**
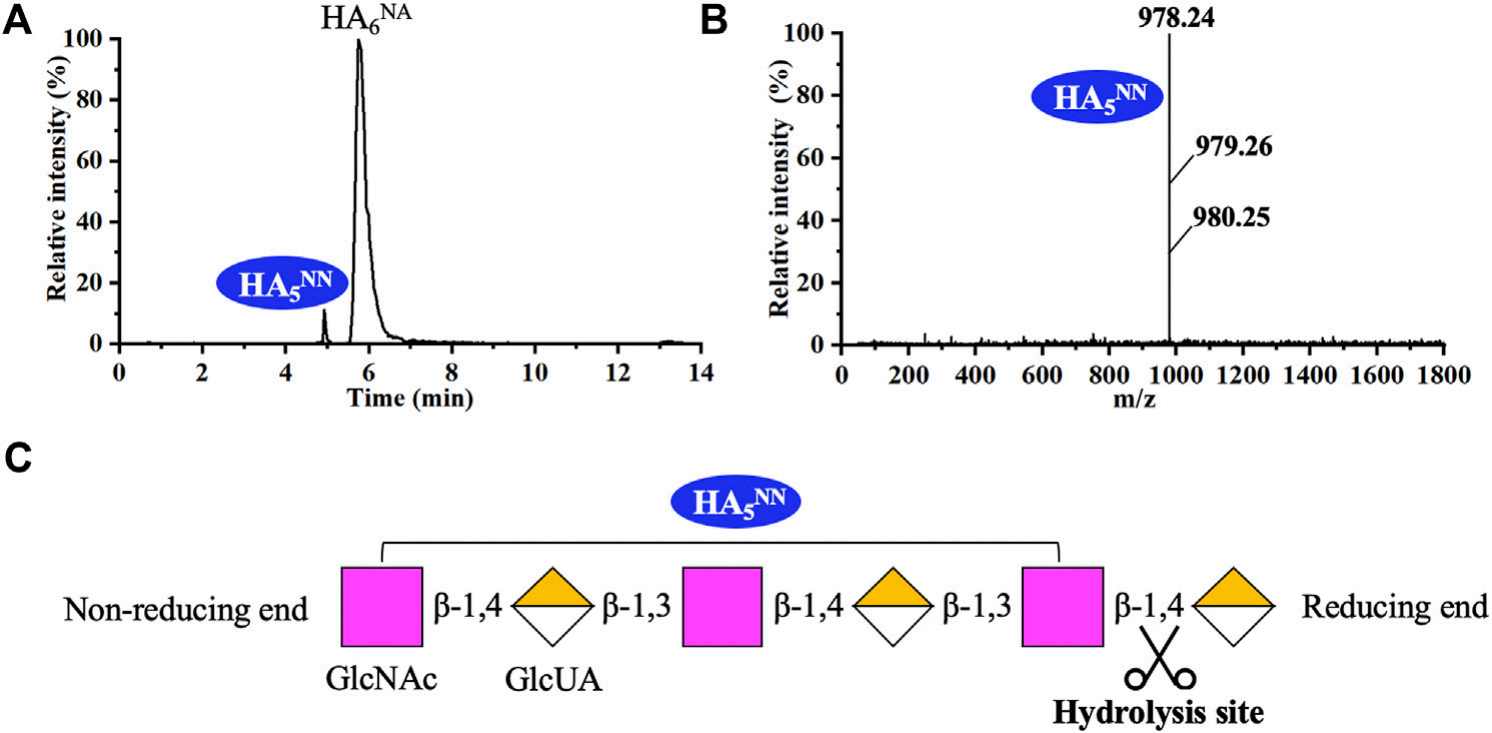
Analysis of the end products of HA_6_^NA^ hydrolyzed by recombinant hPH20. **(A,B)** Extracted ion chromatogram/mass spectra of end products of HA_6_^NA^ by HPLC-MS. **(C)** Schematic diagram of the hydrolysis process of HA_6_^NA^.

**FIGURE 6:**
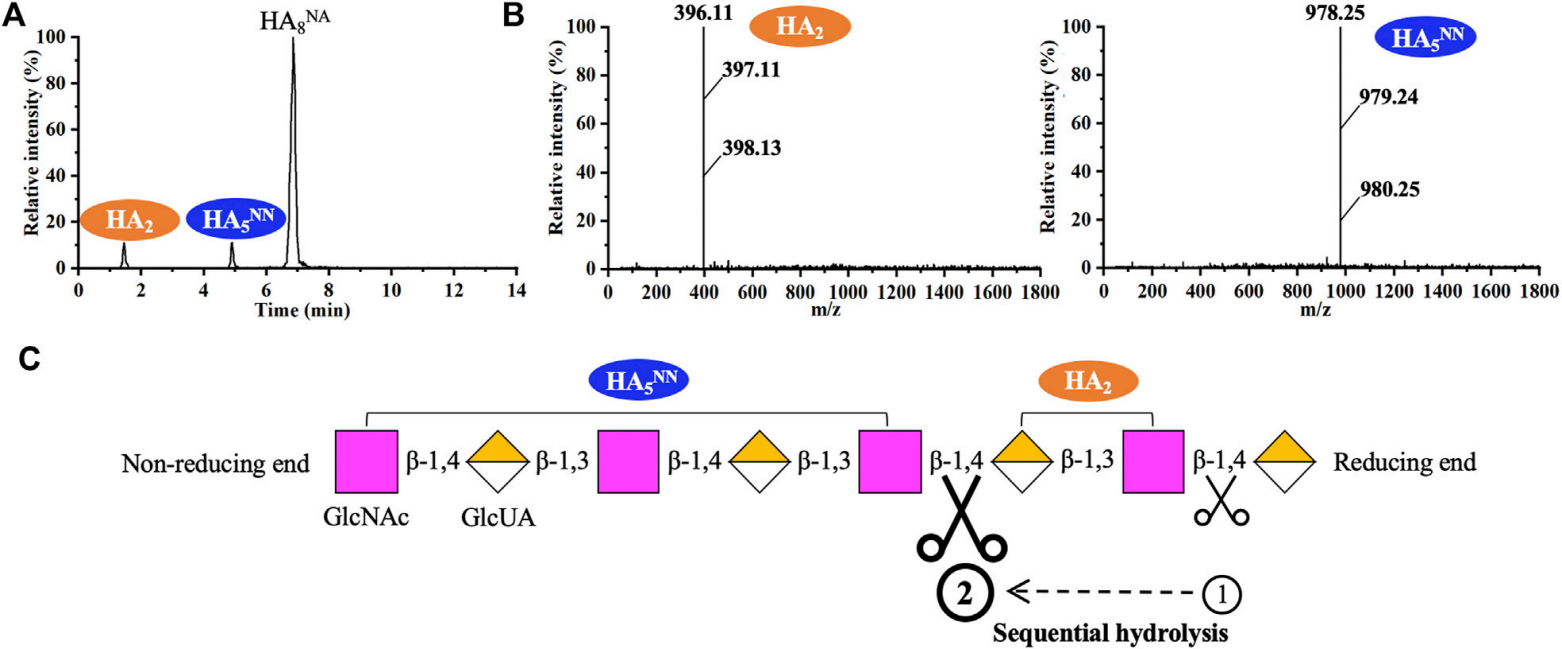
Analysis of the end products of HA_8_^NA^ hydrolyzed by recombinant hPH20. **(A,B)** Extracted ion chromatogram/mass spectra of end products of HA_8_^NA^ by HPLC-MS. **(C)** Schematic diagram of the hydrolysis process of HA_8_^NA^.

As shown in Figure 5, recombinant hPH20 hydrolyzed HA_6_^NA^ producing HA_5_^NN^ as the sole detectable hydrolysate, indicating the enzyme hydrolyzed the β-1,4 glycosidic bond at the reducing end of HA_6_^NA^, while HA_5_^NN^ could not be further cleaved. When using HA_8_^NA^ as the substrate, HA_5_^NN^ and HA_2_ were the main products, while HA_3_^AA^ and HA_7_^NN^ were not detected (Figures 6A,B). The result suggested that recombinant hPH20 hydrolyzed the β-1,4 glycosidic bonds sequentially from the reducing end of HA_8_^NA^, and the hydrolysis capacity toward the penultimate β-1,4 glycosidic bond from the reducing end was higher than that of the β-1,4 glycosidic bond at the reducing end (Figure 6C). In addition, it was noteworthy that recombinant hPH20 could not degrade the β-1,4 glycosidic bond of HA_4_^NA^ (data not shown). From the aforementioned results, we could conclude that recombinant hPH20 hydrolyzed the β-1,4 glycosidic bonds sequentially from the reducing end of o-HAs with HA_6_^NA^ as the smallest substrate. The hydrolysis pattern of recombinant hPH20 differed from BTH, which hydrolyzed the β-1,4 glycosidic bonds from the nonreducing end of o-HAs (Takagaki et al., 1994). A study on the hydrolysis pattern of recombinant hPH20 was of great significance for the directed evolution of the enzyme to prepare a variety of multifunctional o-HAs with specific molecular weights in the future.

## Conclusion

In the present study, human hyaluronidase PH20 was engineered and expressed with high activity in *P. pastoris*. By simplifying the C-terminal domain and introducing protein fusion tags, a fusion mutant AP_2_-△491C was constructed, which showed the extracellular hyaluronidase activity of 258.1 U·L^−1^ in a 3-L bioreactor. To the best of our knowledge, this was the highest reported value of human hyaluronidase PH20. Moreover, our results found that recombinant hPH20 hydrolyzes the β-1,4 glycosidic bonds sequentially from the reducing end of the hyaluronan polysaccharide chain. The smallest substrate for recognition and digestion was HA_6_^NA^.

## Data Availability

The original contributions presented in the study are included in the article/Supplementary Material, further inquiries can be directed to the corresponding author.
