# Supplementary material for: Active Expression of Human Hyaluronidase PH20 and Characterization of Its Hydrolysis Pattern
Source: Front Bioeng Biotechnol. 2022 May 13;10:885888. doi: 10.3389/fbioe.2022.885888 (PMC9136067; doi:10.3389/fbioe.2022.885888)
Supplement: Supplementary file 1 [file DataSheet1.doc]

**Supplementary material**

**Active Expression of Human Hyaluronidase PH20 and Characterization of Its Hydrolysis Pattern**

***Bo Pang^1,2,3^******, Jing He******^2,3^, Weijiao Zhang^2,3^, Hao Huang^2,3^, Yang Wang^2,3^, Miao Wang^1^, Guocheng Du^2,3^ and Zhen Kang^2,3*^***

*^1^School of Food Science and Technology, Jiangnan University, Wuxi, China.*

*^2^The Key Laboratory of Carbohydrate Chemistry and Biotechnology, Ministry of Education, Jiangnan University, Wuxi, China.*

*^3^The Science Center for Future Foods, Jiangnan University, Wuxi, China.*

*Corresponding author. Tel. & fax: +86-510-85918307

*E-mail address:* zkang@jiangnan.edu.cn

**Table S1** Primers used to construct truncated mutants of recombinant hPH20 and add a His×6 tag.

| Primer | Sequence |
| --- | --- |
| △507C-F | CATCATCATCATCATCATTGAGCGGC |
| △507C-R | TGAAATACCTTGGTCCCACACCTCTAACCT |
| △502C-R | CCACACCTCTAACCTCCAGATGAACATAGTG |
| △497C-R | CCAGATGAACATAGTGGCTGATAAAGTGGAG |
| △491C-R | TGATAAAGTGGAGGGTGAAGCGTTATAAAAG |
| △489C-R | AGTGGAGGGTGAAGCGTTATAAAAGATTTGAG |
| △487C-R | GGGTGAAGCGTTATAAAAGATTTGAGGTTCTTCG |
| △485C-R | AGCGTTATAAAAGATTTGAGGTTCTTCGGTC |
| △484C-R | GTTATAAAAGATTTGAGGTTCTTCGGTCTCCATAG |

**Figure S1**

**
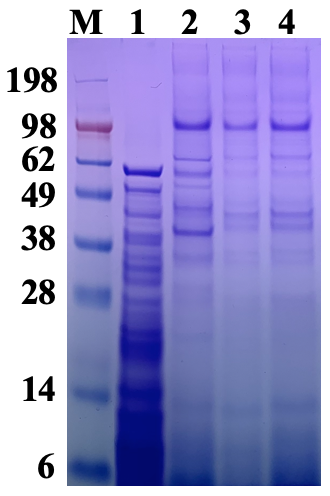
**

**Figure S1.** SDS-PAGE analysis of the expression of recombinant hPH20 and truncated mutants in culture broth. M: standard protein marker; 1: control; 2: recombinant hPH20; 3: △484C; 4: △491C.

**Figure S2**

**
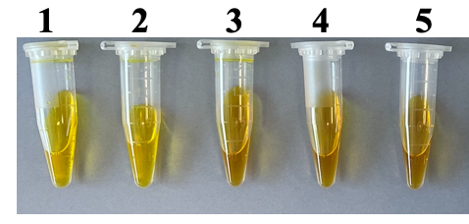
**

**Figure S2.** Hydrolysis of HA by the whole-cell of *P. pastoris* GS115-pPIC9K-*hPH20*. 1: inactivated whole-cell; 2: whole-cell of *P. pastoris* GS115-pPIC9K; 3, 4, 5: whole-cell of *P. pastoris* GS115-pPIC9K-*hPH20*.

**Figure S3**

**
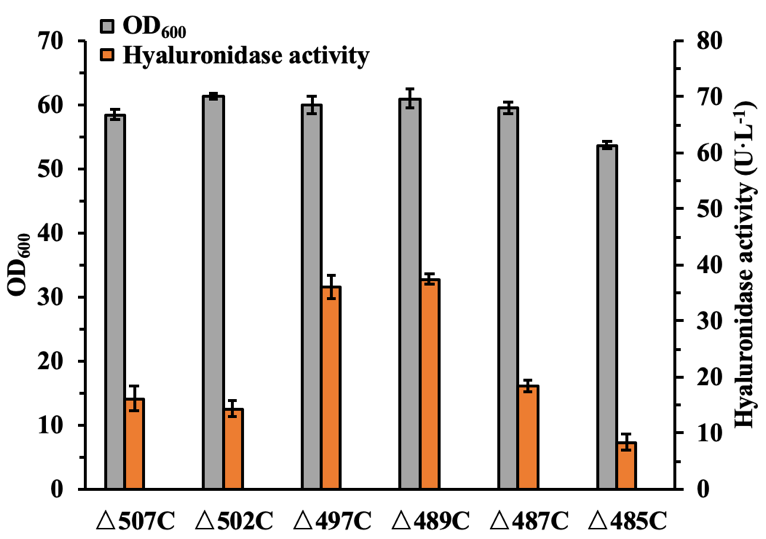
**

**Figure S3.** Hyaluronidase activity of truncated mutants (△507C △502C △497C △489C △487C △485C) in the culture broth.

**Figure S4**

**
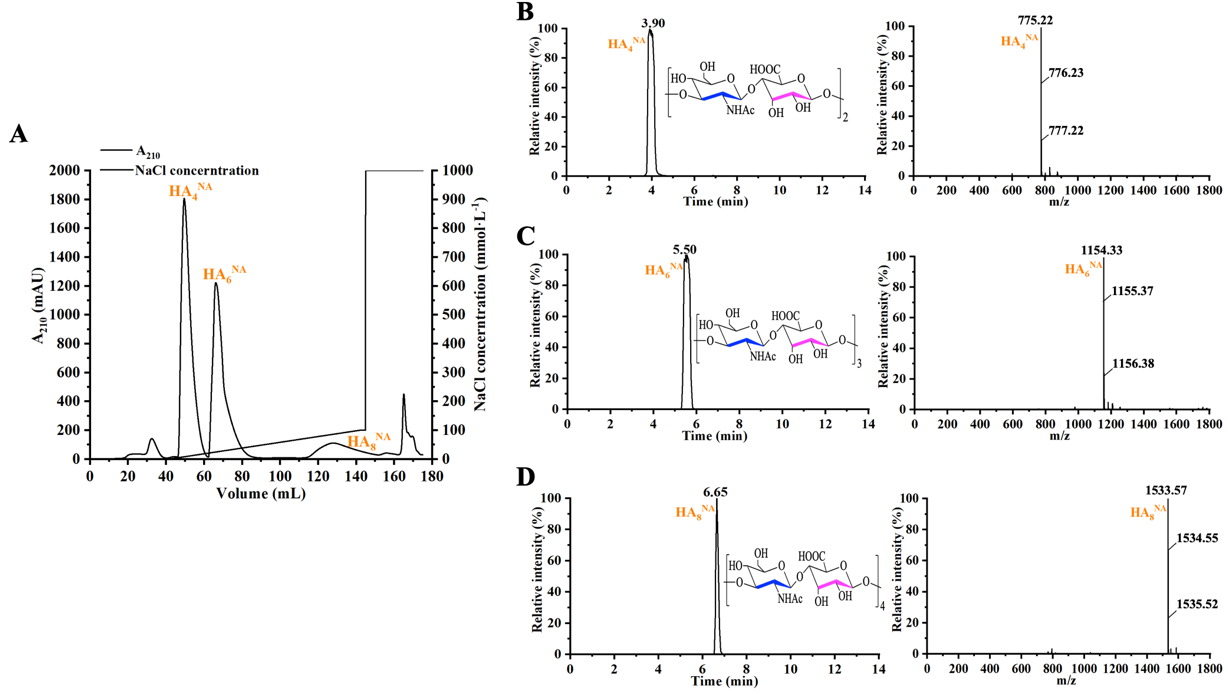
**

**Figure S4.** Separation and identification of HA_2n_^NA^ o-HAs. (A) Anion-exchange chromatograms of HA_2n_^NA^ o-HAs linearly eluted with 0-100 mM NaCl on a Q HP column. (B)/(C)/(D) Ion chromatogram and mass spectra of HA_4_^NA^/HA_6_^NA^/HA_8_^NA^ analyzed by high performance liquid chromatography-mass spectrometry (HPLC-MS).
